# Cognitive and Neurophysiological Recovery Following Electroconvulsive Therapy: A Study Protocol

**DOI:** 10.3389/fpsyt.2018.00171

**Published:** 2018-05-14

**Authors:** Ben J. A. Palanca, Hannah R. Maybrier, Angela M. Mickle, Nuri B. Farber, R. Edward Hogan, Emma R. Trammel, J. Wylie Spencer, Donald D. Bohnenkamp, Troy S. Wildes, ShiNung Ching, Eric Lenze, Mathias Basner, Max B. Kelz, Michael S. Avidan

**Affiliations:** ^1^Department of Anesthesiology, Washington University School of Medicine in St. Louis, St Louis, MO, United States; ^2^Division of Biology and Biomedical Sciences, Washington University School of Medicine in St. Louis, St Louis, MO, United States; ^3^Department of Psychiatry, Washington University School of Medicine in St. Louis, St Louis, MO, United States; ^4^Department of Neurology, Washington University School of Medicine in St. Louis, St Louis, MO, United States; ^5^Department of Electrical Systems and Engineering, Washington University, St Louis, MO, United States; ^6^Department of Psychiatry, University of Pennsylvania Perelman School of Medicine, Philadelphia, PA, United States; ^7^Department of Anesthesiology, University of Pennsylvania Perelman School of Medicine, Philadelphia, PA, United States; ^8^Department of Surgery, Washington University School of Medicine in St. Louis, St Louis, MO, United States

**Keywords:** electroconvulsive therapy, electroencephalography, major depressive disorder, ketamine, anesthesia, seizures, neurocognitive disorders, consciousness

## Abstract

Electroconvulsive therapy (ECT) employs the elective induction of generalizes seizures as a potent treatment for severe psychiatric illness. As such, ECT provides an opportunity to rigorously study the recovery of consciousness, reconstitution of cognition, and electroencephalographic (EEG) activity following seizures. Fifteen patients with major depressive disorder refractory to pharmacologic therapy will be enrolled (Clinicaltrials.gov, NCT02761330). Adequate seizure duration will be confirmed following right unilateral ECT under etomidate anesthesia. Patients will then undergo randomization for the order in which they will receive three sequential treatments: etomidate + ECT, ketamine + ECT, and ketamine + sham ECT. Sessions will be repeated in the same sequence for a total of six treatments. Before each session, sensorimotor speed, working memory, and executive function will be assessed through a standardized cognitive test battery. After each treatment, the return of purposeful responsiveness to verbal command will be determined. At this point, serial cognitive assessments will begin using the same standardized test battery. The presence of delirium and changes in depression severity will also be ascertained. Sixty-four channel EEG will be acquired throughout baseline, ictal, and postictal epochs. Mixed-effects models will correlate the trajectories of cognitive recovery, clinical outcomes, and EEG metrics over time. This innovative research design will answer whether: (1) time to return of responsiveness will be prolonged with ketamine + ECT compared with ketamine + sham ECT; (2) time of restoration to baseline function in each cognitive domain will take longer after ketamine + ECT than after ketamine + sham ECT; (3) postictal delirium is associated with delayed restoration of baseline function in all cognitive domains; and (4) the sequence of reconstitution of cognitive domains following the three treatments in this study is similar to that occurring after an isoflurane general anesthetic (NCT01911195). Sub-studies will assess the relationships of cognitive recovery to the EEG preceding, concurrent, and following individual ECT sessions. Overall, this study will lead the development of biomarkers for tailoring the cogno-affective recovery of patients undergoing ECT.

## Introduction

### Seizures—unique states for probing the return consciousness and cognition

The return of consciousness following reversible states of unresponsiveness is relevant to neuroscience and clinical practice. Neural mechanisms underlying these processes appear to be distinct, with implications for anesthetic practice (1) and sleep/wake disorders (2). While states incurred by general anesthesia (3–5) and sleep (6, 7) have suggested neural substrates necessary for sustaining consciousness (8), the recovery from these depressed states of neural activity remains poorly characterized. Comparatively less is known regarding the recovery from states of highly synchronized neural activity incurred through generalized seizures (9, 10). Characterizing the recovery of neural activity and cognitive function following these states may provide a system to complement states of brain suppression given that: (1) action potential synchronization is a fundamental mode of information processing in the cerebral cortex distinct from neuronal firing rates and (2) seizures arise from changes in the excitatory and inhibitory synaptic balance in different brain regions.

The relationships of underlying electroencephalographic (EEG) activity and the recovery from generalized seizures is currently limited and challenging to investigate (11). Generalized seizures are characterized by the loss of consciousness coincident with ictal EEG spike-and-wave complexes, polyspike-and-wave complexes, and spikes (12). External phenotypes, ranging from convulsions to immobile staring, likely depend on the precise disruption in sub-cortical arousal systems, cortical-subcortical interactions, or neocortical connectivity (9, 10, 13). Inter-individual heterogeneity among clinical seizures may arise from diverse structural or metabolic derangements. Moreover, seizures are typically sporadic, unpredictable in occurrence, and may vary in intensity and character, making them difficult to study systematically. Following the disappearance of epileptiform EEG signatures, the postictal period begins and culminates in a return of consciousness and cognitive function. There is little standardization of postictal clinical and behavioral testing to facilitate objective comparison to EEG changes during the postictal period. Critical barriers to generating inferences from reproducible seizures may be addressed in the context of electroconvulsive therapy (ECT) (14), where seizures are electrically induced under safely controlled conditions.

### EEG activity and cognitive dysfunction following ECT

The potential of EEG to inform clinicians on the future efficacy and side effects of ECT has not been fully realized. EEG is commonly monitored during ECT, a proven treatment for depression, bipolar illness, and psychosis (15). Following the delivery of the ECT stimulus charge, epileptiform activity in the bilateral fronto-mastoid EEG can complement the assessment of peripheral tonic-clonic muscle activity (16). Clinically relevant EEG measures beyond the length of seizure duration (17) have unclear clinical utility (18). Optimally, EEG markers would be available for predicting and refining ECT administration to balance efficacy and side effects that accrue over the course of therapy. EEG measurements acquired on the day of treatment would inform clinicians prior to stimulus delivery. The spatial, temporal, and spectral properties of such markers remain unknown. Once ascertained, translation to clinical practice would require a sparse montage of EEG sensors that can generalize across patient gender, age, and recovery from general anesthesia. Similar advances in the field have not occurred since prior work establishing a relationship between cognitive performance and ECT stimulation parameters (19, 20). This void may be due to the paucity of studies that have characterized EEG across widely distributed brain regions using either 10–20 montages (21–24) or high-density EEG. The spatial resolution offered by high-density EEG is likely needed to associate cognitive and affective perturbations to specific EEG patterns. High-density scalp EEG has shown superiority over standard 10–20 montage recordings in guiding surgical treatment for epileptic seizures (25). Extension of this paradigm to the ECT setting may yield clinically relevant EEG markers for tailoring treatment at an individual patient level.

During the ictal period, seemingly stereotyped EEG patterns develop and resolve (26), with proposed phases of activity (21). Brief periods of EEG suppression or rhythmic bilateral 14-22 Hz oscillations may first emerge (21). Induction of bilateral poly-spike activity occurs, often with greater power on the side of the stimulation electrode in unilateral ECT (21, 27). Spike/spike-and-wave complexes then arise, followed by termination within 3 min (21, 26). This ictal activity may be followed by postictal generalized EEG suppression, a marker associated with ECT therapeutic efficacy (17, 28, 29) and with potential implications for understanding sudden unexpected death in epilepsy (30, 31). Delta waves (<4 Hz) (26) emerge that are gradually replaced by theta (4–8 Hz), alpha (8–13 Hz), and beta (13–30 Hz) rhythms (26). The dynamics of these EEG changes may aid in our understanding of seizures in general and help to reveal the efficacy/side effects of ECT in the postictal period following future large-scale investigations.

The continued resolution of postictal EEG changes may also correlate with the cognitive impairments incurred immediately after individual sessions and progress over the course of ECT treatments. Perturbations in processed EEG measures persist even when individuals appear to be awake in the postictal period (32–34). Patients with greater suppression in processed EEG measures following ECT are more likely to experience prolonged memory impairment (35). Specific EEG markers that can be linked to both underlying neurobiology and cognitive function have not been developed for the acute period after ECT. Beyond individual sessions, persistent slow theta and delta oscillations have been observed in the EEG (36) and may resolve only weeks following the last session (37, 38). These markers that remain weeks after ECT sessions may be linked to either therapeutic efficacy (38) or the extent of disorientation and retrograde amnesia (39). Definitive relationships remain speculative.

The recovery of cognitive function following individual ECT sessions has not been fully characterized. Recent analyses have shed light on the incidence and persistence of cognitive side effects related to this procedure (40). Approximately 5–12% of patients experience postictal agitation and disorientation after ECT (41, 42) that may last 1–2 h after ECT. Postictal agitation does not occur reproducibly in the same patients following subsequent treatments (43). Disorientation during the early postictal recovery from ECT appears to decrease with number of sessions (44). In contrast, interictal confusion accumulates with successive ECT sessions (44). Cognitive side effects may persist for 90 min following seizure termination (45). The temporal development and progression over the course of ECT remain unclear, but deficits in verbal memory, executive function (46), and visuospatial memory (47) have been identified. Cognitive impairments during the course of ECT can delay treatment (48), contribute to missed work, burden caregivers. Given that each session of ECT requires the administration of general anesthesia during seizure induction, systematic study requires accounting for these potent neuromodulatory agents.

### Control for anesthetic exposure

Elucidating cognitive recovery following seizures in the context of ECT requires a control to account for the effects of general anesthesia. This is because modern ECT is conducted under general anesthesia and pharmacologic neuromuscular paralysis. Anesthetics with mechanisms invoking γ-aminobutyric acid (GABA) A-type receptor agonism (e.g., etomidate) or NMDA-receptor antagonism (ketamine) are commonly used. Ketamine has received greater attention recently since subanesthetic doses of ketamine have shown efficacy in treating refractory depression (49–52). Relative to other anesthetics in use for ECT, ketamine may provide faster recovery from cognitive impairment on the day of treatment (53, 54) or offer additive benefits on ECT efficacy (54). Thus, ketamine may be useful in a sham ECT condition to control for anesthetic exposure while offering potential therapeutic effects even in the absence of electrical stimulation.

### Hypotheses and aims

This is a randomized crossover trial to investigate the recovery of cognitive and neurophysiological function following right-unilateral ECT in individuals with treatment-resistant depression. We hypothesize that the reconstitution among different cognitive domains will markedly vary in rate and order, depending on the presence of seizures induced by electrical brain stimulation. Our specific aims include: (1) assess whether the time to return of responsiveness will be prolonged with ketamine + ECT compared with ketamine + sham ECT; (2) ascertain whether the time of restoration to baseline function in each cognitive domain will take longer after ketamine + ECT than after ketamine + sham ECT; (3) determine if postictal delirium is associated with delayed restoration of baseline function in all cognitive domains; and (4) determine whether the sequence of reconstitution across cognitive domains is similar to that occurring after an isoflurane general anesthetic; we also anticipate these cognitive disturbances to mirror recovery in EEG power spectral measures, despite substantial variability across our sample.

## Methods and analysis

This protocol includes elements elaborated in the Standard Protocol Items: Recommendations for Interventional Trials (SPIRIT) checklist (55, 56).

### Participants

#### Ethics approval

The HRPO at Washington University School in St. Louis has approved the study. The study will be conducted with strict adherence to Washington University Institutional Review Board protocol. American Board of Anesthesiology board-certified anesthesiologists with experience in conducting clinical studies will lead the study. Safety and privacy of study participants will be safeguarded in compliance with the Health Insurance Portability and Accountability Act.

#### Inclusion and exclusion criteria

Inclusion criteria include: (1) Referral for ECT via right unilateral stimulation for treatment-resistant non-psychotic unipolar depression or bipolar disorder, (2) Fluency in English, (3) Age greater than 18 years, and (4) Ability to provide written informed consent. Exclusion criteria are: (1) Known brain lesions or neurological illness with sufficient cognitive impairment to prevent cognitive testing prior to ECT initiation, (2) Schizophrenia, (3) Schizoaffective disorder, (4) Blindness or deafness that may impair performance on cognitive testing, or (5) Inadequate seizure duration with etomidate general anesthesia, defined at our institution as bilateral spike-and-wave complexes present for less than 10 s. Past suicidality or substance use disorder will not exclude patients from enrollment.

### Design and procedure

#### Recruitment

Given the sample size used in our prior study (57), 15 patients will be recruited over a span of three years. Psychiatrists will brief prospective patients on the study to determine eligibility and interest. Trained study team members will formally screen and enroll interested patients during a preoperative clinic visit or in the hospital ward. All enrolled patients will provide written informed consent, with adherence to the Declaration of Helsinki. Patients will not be charged for participating and will receive remuneration of $100 per completed treatment session, up to $600.

#### Interventions

**Figure 1:**
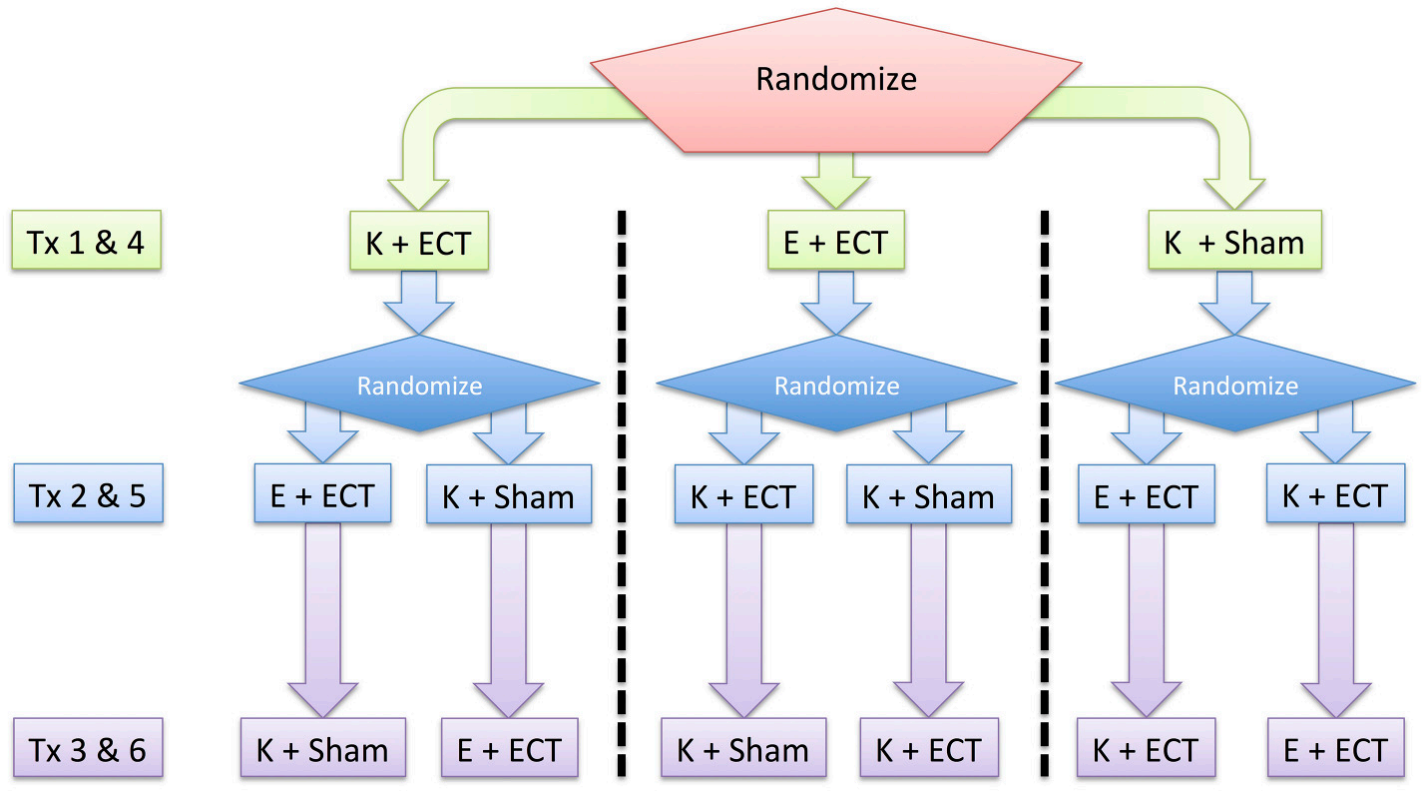
Overall study design. Patients are randomized to repeated testing on three different treatment (Tx) interventions: Etomidate + ECT (E + ECT), ketamine + ECT (K + ECT), and ketamine + sham ECT (K + Sham). These interventions occur over a 2-week period following a dose-charge titration session that determines the adequate ECT charge for treatment-resistant depression.

Each patient will be scheduled for an initial dose-charge titration and six treatment sessions over the initial 2 weeks of the ECT treatment cycle (Figure 1) at Barnes-Jewish Hospital, St. Louis, MO, USA. Patients will receive three sequential treatments: etomidate + ECT, ketamine + ECT, and ketamine + sham ECT. These treatments will be repeated in the same sequence over the subsequent week. The study focuses on changes in in cognoaffective function over the initial period of the ECT index course prior to maintenance therapy. A 2-week duration of involvement was chosen to maximize patient tolerance of study procedures and minimize repetition of stimuli.

### Randomization and blinding

A trained team member will use a computer-generated randomization algorithm among 18 potential combinations of initial cognitive task and order of study interventions. Random assignments will account for investigator-physician availability for ketamine + sham ECT sessions. The patient will be blind to the order of treatment condition. Study and clinician teams will be aware of the treatment arm at each session and maintain routine checks and monitoring before and after anesthetic induction. In order to maximize adherence to the intervention protocol, a study coordinator will inform research and clinical teams of the treatment condition prior to the study session day, and brief these teams on study procedures immediately before the subject's scheduled treatment session. To maintain sufficient blinding of the subject to the treatment condition, patients will not be able to view the syringe during anesthetic induction during any treatment session. Furthermore, stimulation electrode and conducting gel will also be placed on the scalp following loss and prior to return of responsiveness during sham-ECT sessions. Post-anesthetic evaluation by anesthesiology, psychiatry, and nursing staff will be consistent across sessions.

To maximize study rigor and reproducibility, investigators evaluating study measurements for data quality and development of analytical tools will be blinded to the details of the treatment intervention, whenever possible (58).

### Timeline of treatment visits

#### Dose-charge titration

**Figure 2:**
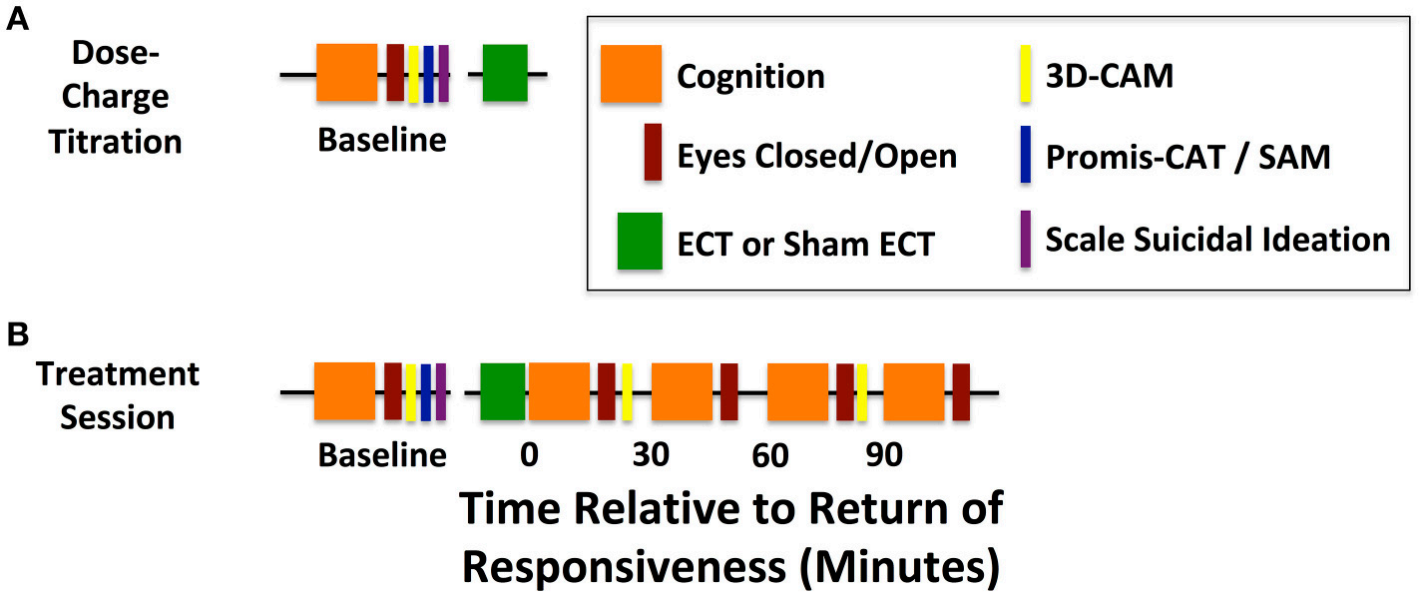
Timelines for dose-charge titration and treatment sessions**. (A)** Baseline cognitive assessments are acquired before the dose-charge titration session with etomidate general anesthesia. **(B)** Baseline cognitive assessments are acquired before each treatment session and serially from the return of responsiveness to verbal command (T = 0) and up to 90 min afterwards (T = 90). Continuous high-density EEG is acquired during baseline assessments and treatment sessions.

As part of standard care, the patient will be admitted for an initial dose-charge titration to induce a generalized seizure of adequate duration under etomidate general anesthesia. This session will serve to determine the seizure threshold and tolerance for the study procedures (Figure 2A). During this visit, the study team will assess baseline cognitive function and EEG prior to ECT, tolerability of cognitive testing and EEG recording, and feasibility of etomidate as the anesthetic for study and subsequent treatments. ECT charge will be delivered via a Thymatron System IV (Somatics, LLC, Venice, FL, USA). Per ECT laboratory procedures, stimulation parameters include a current of 0.9 amperes, pulse width of 0.3 ms, with escalating dosage: (5% total charge: 24.9 millicoulombs, 10 Hz stimulation, 4.6 s duration; 10% total charge: 50.8 millicoulombs, 20 Hz stimulation, 4.65 s duration, 15% total charge: 75.6 millicoulombs, 20 Hz stimulation, 6.98 s duration). Subsequent planned ECT treatment is based on a six-fold increase in charge delivery. Thus, individuals without suitable seizure duration at a projected 100% charge will be withdrawn from the study if subsequently scheduled for bilateral ECT. Participants will not be withdrawn if their standard-of-care anesthetic is changed from etomidate to another anesthetic, such as ketamine.

#### Study intervention treatment sessions 1–6

Following randomization, each patient will initiate a complete course of therapy with participation in six treatment sessions (Figure 2B). During four of these six sessions, the patients will receive care within standard practice for ECT: general anesthesia (etomidate or ketamine), muscle paralysis (succinylcholine), and electrical stimulation. For the remaining two sessions, patients will undergo a ketamine general anesthetic without muscle paralysis or ECT stimulation.

High-density EEG, bilateral fronto-mastoid clinical EEG, and full American Society of Anesthesiologists (ASA) monitoring will commence prior to the induction of general anesthesia. Additionally, audible squeeze toys will be placed in each hand of the patient, who will be instructed to follow serial commands to either “Squeeze your left hand twice” or “Squeeze your right hand twice.” Every 30 s, one of these recorded audio commands will be played at random, to monitor loss and return of responsiveness to verbal command. Patients will be pre-oxygenated by mask and anesthesia will be induced with ketamine, approximately 2 mg/kg, or etomidate, approximately 0.2 mg/kg. Following bolus of the induction agent, loss of responsiveness and eyelash reflex will be confirmed. Loss of responsiveness to verbal command will be recorded as the first time when a subject fails to correctly respond to the standardized auditory commands. Care adherent to the ASA guidelines will be performed regardless of treatment session. For sessions with ECT, pre-stimulation hyperventilation and assisted ventilation will also be performed. Central seizure duration will be assessed from bilateral fronto-mastoid EEG by the psychiatry team. Peripheral seizure duration will be determined through monitoring of tonic-clonic activity (16). During all sessions other drugs will also be administered according to current practice and as clinically indicated (e.g. for nausea or headache).

During the recovery from the study intervention, the study team will note spontaneous eye opening and compliance to commands to ascertain the timing for return of responsiveness. The ability to extend the thumb to verbal command (“thumbs up”) will be assayed every 30 s. Timing for opening of eyes to command will be noted. These requests will be preceded with the patient's name to increase emotional valence. Return of responsiveness will be defined as the first time at which the patient squeezes the correct hand precisely as instructed via the standardized auditory commands. At this time, defined as t = 0 min, the subject will begin a series of cognitive and behavioral assessments, which are repeated every 30 min up to 90 min after return of responsiveness. Patients will be permitted to take brief breaks to use the restroom and eat or drink, as necessary. They will be discharged according to standard post-anesthesia care unit discharge criteria upon completing the last neurocognitive test battery. A study site coordinator will contact each subject within 24 h of the study day to assess and document any adverse events, as well as confirm the subject's continued involvement in the study.

## Data collection

The overall design of data collection for the dose-charge titration session and the experimental treatment sessions differ in the emergence period after ECT or sham ECT (Figure 2). Primary study measurements and outcomes are listed in Table 1. Patient demographics and clinical measures will be maintained using the Research Electronic Data Capture (REDCap) application (59).

**Table 1:** Summary of study measurements and outcomes for primary analyses.

**Type**	**Measure/Outcome**	**Purpose**
Responsiveness	Spontaneous eye opening	Spontaneous return of arousal
	Eye opening to verbal command	Verbal probe
	Mimicking “thumbs up” gesture	Probe with visual cues
	Squeezing of hand to verbal command	Standardized auditory probe
Cognitive Task Performance	Motor Praxis Test	Sensorimotor speed
	Psychomotor Vigilance Test	Reaction time
	Digit Symbol Substitution	Visual scanning
	Fractal-2-Back	Working memory
	Visual Object Learning Test	Memory for complex shapes
	Abstract Matching Test	Executive function
Clinical Outcomes	Scale of Suicidal Ideation	Suicidal Ideation
	PROMIS-CAT	Depression: Computer-based
	Self-Assessment Manikin	Depression: Visual-analog
	3D-CAM	Delirium Assessment
	QIDS-SR16	Depression: Self-report
Seizure Markers	Frequency	Rate of ictal complexes
	Scalp Topology	Spatial characteristics
	Morphology	Temporal characteristics
	Postictal Generalized EEG Suppression	Efficacy marker
Power Spectral Measures	Delta Band (1–4 Hz)	
	Theta Band (4–8 Hz)	
	Alpha Band (8–13 Hz)	Spectral content
	Beta Band (13–30 Hz)	
	Gamma Band (30–70 Hz)	

### Primary and secondary outcomes

Primary outcomes include: the temporal recovery profiles for cognitive task performance, as measured using the Cognition assessment battery (60); times for the return of responsiveness to auditory command; and the presence of delirium, evaluated through the 3-minute Diagnostic Assessment for CAM-defined delirium (3D-CAM) (61).

Secondary outcome measures based on the EEG will include characterization of the seizures by both expert reader interpretation and quantitative techniques (62). Central seizure duration will be visually determined by clinician evaluation of the frontal-mastoid bipolar EEG ictal complexes. Additional measures will be calculated from windowed analyses of the high-density EEG: power spectral measures of the interval between anesthetic induction to delivery of ECT stimulus charge; the seizure envelope for 1–12 Hz EEG power; peak-to-peak amplitude, calculated from the difference in maximum and minimum voltages within 200 ms time; periodicity of epileptiform discharges; inter-hemispheric symmetry of seizure discharges; intra- and inter-hemispheric coherence. When possible, these measures and power spectral estimates will be derived from different phases of the ictal waveforms (27). The following postictal EEG measures will be assessed: duration and signal amplitude of postictal EEG suppression; power spectral parameters flanking the return of responsiveness to verbal command; and power spectral parameters from eyes open and closed epochs, including assessments of the posterior dominant rhythm. Spatiotemporal analyses will also focus on the propagation of EEG signatures during the ictal and early postictal period.

Additional secondary outcomes include: suicidality, mood, depression severity, delirium, and treatment course outcome.

### Cognition test battery

Cognitive assessments will be administered on a Dell Latitude E5430 Laptop with a 14-inch liquid crystal display (Round Rock, TX, USA) using Cognition test battery software (60). Cognition consists of 10 brief neuropsychological tests with known cerebral representation that cover a wide range of cognitive domains (60). Patients will watch a standardized instructional video for baseline testing prior to the dose-charge titration session. Additional pre-intervention baseline assessments will also be performed on each treatment session prior to induction of general anesthesia. Post-treatment testing will occur at return of responsiveness to auditory command (*t* = 0) and at *t* = 30, 60, and 90 min. Each testing bout will take approximately 15–20 min to complete wherein the first cognitive test will be repeated as the last test at each time point. The order in which the tests are administered will be randomized between subjects but will be held constant across treatment sessions for a given patient. These tests have been previously employed in probing cognitive function during the recovery from isoflurane general anesthesia (57).

#### Motor praxis test (MPT)

This test assesses sensorimotor speed (63). Participants use a touchpad to click on squares that appear at random locations on the screen. The difficulty of tracking increases as successive squares become smaller.

#### Psychomotor vigilance test (PVT)

The PVT evaluates reaction time for detecting visual stimuli at random inter-stimulus intervals (64). During this 3-min test, subjects are instructed to monitor for a counter on the screen and to hit the space bar as quickly as possible once the counter appears.

#### Digit symbol substitution test (DSST)

This test assesses memory, complex scanning, and visual tracking based on a paradigm used in the Wechsler Adult Intelligence Scale (65). Subjects are presented with a legend that pairs unique symbols to digits (1 through 9). Symbols are then sequentially presented on the screen in random order over a 90 s testing period. Participants are instructed to press the corresponding number key as soon as possible.

#### Fractal-2-back (F2B)

As a variant of the Letter 2-Back task (66), the F2B assesses working memory through sequential presentation of fractal patterns that fill the screen. During the testing bout, each pattern may have multiple presentations. Subjects are asked to press the space bar when the current stimulus matches the pattern displayed two fractals previously.

#### Visual object learning test (VOLT)

Memory for complex visual figures is evaluated by the VOLT (67). Ten complex three-dimensional figures are presented on the screen for participants to memorize. Participants are then asked to select these ten objects from a set that also contains ten decoys, with each of the 20 objects presented individually in random order.

#### Abstract matching test (AMT)

This test assesses components of executive function, including the development of implicit abstract rules (68). During the AMT, two pairs of objects are shown, one at the bottom left, and one at the bottom right of the screen. A target object appearing in the middle of the screen must be classified as fitting better with one of the two groups based on shape or fill pattern.

MPT, PVT, and DSST use stimuli that are randomly generated prior to each test administration. For F2B, VOLT, and AMT, 15 unique versions exist, and up to 14 versions will be used for this protocol. The same stimuli will be repeated after the 14 versions of these tests have been exhausted. Due to the number of stimuli and the time between ECT sessions, it is unlikely that subjects will remember specific stimulus sequences or stimuli across ECT sessions.

Median reaction time and accuracy will be computed for each of the multiple testing bouts within each session, including pre-treatment baseline and during recovery. For each treatment session, parameters obtained after the return of responsiveness to verbal command will be subtracted from baseline to arrive at repeated measures that account for the recovery in task performance over time.

### Suicidal ideation, depression severity, and delirium assessment

At the beginning of each study session, the first two questions of the Scale of Suicidal Ideation (69) will be used to assess the patient's subjective desire to hurt him- or herself. These two questions, “Wish to live?” and “Wish to die?”, have been used previously for assessing changes in suicidal ideation before and after a brief ketamine infusion for major depressive disorder (70).

Depression severity will be assessed using the Patient-Reported Outcomes Measures Information System-Computer Adaptive Testing (PROMIS®-CAT) survey for depression. Mood will be quantified via the Self-Assessment Manikin (SAM, Supplementary Material 1) (71), a brief scale appropriate for patients with transient cognitive impairment following ECT. Baseline assessments will be performed prior to the dose-charge titration. To determination improvement on the day of treatment, the PROMIS®-CAT and the SAM will be administered prior to anesthetic induction on all study sessions and after the last cognitive battery of each treatment session. As part of standard care for tracking depression symptoms during an ECT course, treating psychiatry teams will administer the Quick Inventory of Depressive Symptomatology, Self-Report (16-Item), QIDS-SR16 (72).

Delirium will be assessed with the 3-min Diagnostic Assessment for CAM-defined delirium (3D-CAM) (61). The 3D-CAM will be administered at baseline, prior to the dose-charge titration visit, prior to each treatment visit, and post-treatment at *t* = 0, and 60. However, if the patient is negative for the 3D-CAM assessment at any time during the recovery, subsequent assessments will not be performed during the remainder of the session.

To assess quality of treatment blinding, the patient will be asked their impression of whether they received ECT at the last testing point on each treatment session. The patient will be asked, “Do you feel that you received ECT today? Was ECT painful?”

### EEG and video acquisition

EEG will be collected during the dose-charge titration session and during all treatment sessions to assess pre-ECT and pre-anesthetic baseline EEG recordings. An appropriately fitted 64-channel EEG Geodesics Sensor Net (Electrical Geodesics, Inc. Eugene, OR, USA) will be affixed to the scalp and face. Elefix electrode paste (Nihon Kohden America, Inc., Irvine, CA, USA) will be injected to maintain conductivity to the silver/silver-chloride electrodes. Electrode impedances on each channel will be optimized to be less than 100 kOhms/channel, per manufacturer's suggestions. EEG signals (500 Hz sampling rate) will be acquired with a Net Amps 400 amplifier and Net Station version 5.0 and above (Electrical Geodesics, Inc. Eugene, OR, USA) via a Late 2012 Mac Pro Workstation (Apple Cupertino, CA, USA). Whenever possible, video synchronized to EEG will be acquired using an Axis P3364LV network camera (Axis Communications, Lund, Sweden).

### EEG preprocessing and analysis

Netstation Tools and EEGLab will be used to assess for quality and to reduce artifact related to motion and eye movements (73). Bad channels will be identified by visual inspection. Signals will be filtered from 1 to 100 Hz and subsequently downsampled to 250 Hz. Modules of the PREP pipeline (74) will be used to reduce artifact related to line noise and movement. Reduction of eye movement artifacts will employ independent component analysis. For ictal recordings, preprocessing will be tailored to minimize distortion of seizure complexes. The following analysis time epochs will be evaluated for secondary outcomes related to EEG activity: pre-ECT and post-ECT periods with either eyes open or eyes closed, the interval between anesthetic induction and ECT stimulation, the period between stimulus delivery and cessation of ictal waveforms/spike-and-wave complexes, postictal period of EEG suppression and slowing, and both 5-min epochs flanking the return of responsiveness to verbal command.

Spectral analysis will be performed using the Chronux Toolbox (75), including five tapers, time frequency bandwidth of 3, and 6-s non-overlapping time windows. Total power and peak amplitude will be computed within the delta (1–4 Hz), theta (4–8 Hz), alpha (8–13 Hz), and beta (13–30 Hz) bands. Sub-studies will focus on coherence and phase lag indices, as computed using the Chronux toolbox. Global coherence (76) and permutation entropy will also be computed (77).

In parallel to power spectral analyses, we will track the time-varying connectivity between the measured brain regions. Data will be windowed into 5–10 s epochs within which several cross-channel connectivity metrics will be computed. These measures will include the Pearson correlation, directed entropy, and Shannon mutual information. Thus, windows will manifest different “networks,” each describing the association between channels (regions) according to its respective metric. These networks will be clustered into a set of distinct motifs, or microstates, by using a k-means algorithm with least-squares error criterion. Other methods to characterize the time-varying dynamics of the observed brain activity, including those based on network control theory, will also be considered.

Visualization and analyses of epileptiform EEG activity will be performed using Net Station and Persyst software (Persyst, Solana Beach, CA, USA), following interpolation of bad channels and re-referencing to the average signal. EEG dipole localization, inter-hemispheric generalization, and phase-reversals will be assessed by epilepsy board-certified neurologists. Seizures will be staged according to the previously described phases that follow ECT stimulation (phase I with initial 14–22 Hz rhythmic beta activity, phase II with arrhythmic polyspike activity, and phase III with rhythmic 2.5–3.5 Hz. spike/polyspike activity 27). The duration and amplitude of post-ictal generalized EEG suppression (PGES) will also be determined. Additionally, expanding on previous analyses of stationarity in epileptiform activity induced by ECT (24), we will use high-density EEG to topographically map rhythmic sharp-wave discharges. To maintain rigor, evaluators will be blinded to the study intervention. Quantitative metrics will be derived from spectral and time-based analyses of the ictal EEG. These measures will evaluate seizure energy, periodicity, and symmetry, as well as propagation, and termination.

### Sample size

We based our targeted enrollment on safety consideration for a ketamine general anesthetic, expected differences in the recovery patterns of different domains assessed by the Cognition test battery, and prior volunteer data with isoflurane emergence (57). Sample size calculations were based on 1-way pairwise of ANOVA (Analysis of Variance) comparisons of 5 means (*t* = 0, 30, 60, 90, and 120 min). Expected effect sizes for differences in the modeled trajectories of cognitive function ranged from 20 to 40 min. Standard deviations were expected to range between 20 and 40 min. Using conservative assumptions, we calculated a sample size of 24 subjects (effect size, μ_A_-μ_B_, of 20 min; standard deviation, σ, of 20 min; two-sided alpha of 0.05; power of 80%). With liberal assumptions, we estimated a sample size of 12 participants (μ_A_-μ_B_ of 40 min; σ of 20 min; two-sided alpha of 0.05; power of 99%). With this range of estimates and expected attrition of participants, we targeted for data collection from 15 to 20 participants.

### Statistical analyses

We will employ mixed-effects models to quantify trajectories of cognitive recovery over time while addressing inter-subject variance and missing data. Linear models with appropriate transformations will be used preferentially over non-linear models. Time and treatment intervention group will be included as fixed effects while random effects will account for repeated measures provided by each participant. Day of treatment relative to the dose-charge titration session will allow consideration of cumulative effects of treatment order that may remain biased despite randomization. To address differences in ECT-stimulation responsiveness, regression approaches will account for dose charge, central seizure duration, and age. Models will assess for the effects of treatment on the timing for the return of responsiveness to verbal command and the presence of delirium. Separate mixed-effects models will be generated to assess task performance in a group of young healthy volunteers during the recovery from isoflurane general anesthesia (57). For example, the following damped-exponential equations will be used for mixed-effects models of cognitive task performance over time *t*, for an individual *k*.

(1) $$\begin{array}{l} \text{Reaction Time }\left(\text{t},\text{k}\right)\;=\;\Phi_{1}\left(\text{t},\text{k}\right)-\Phi_{2}\left(\text{t},\text{k}\right)\;\times \text{ exp}\left(-\Phi_{3}\left(\text{t},\text{k}\right)\right) \end{array}$$

(2) $$\begin{array}{l} \text{Accuracy }\left(\text{t},\text{k}\right)\;=\;\Phi_{1}\left(\text{t},\text{k}\right)+\Phi_{2}\left(\text{t},\text{k}\right)\;\times \text{ exp}\left(-\Phi_{3}\left(\text{t},\text{k}\right)\right) \end{array}$$

Where parameters Φ_1_(*t*,*k*), Φ_2_(*t*,*k*), Φ_3_(*t*,*k)* are optimized at an individual and group level based on changes in these measures relative to pre-intervention baseline within a treatment session. A change in performance at time 0 (return of responsiveness) would be accounted by Φ_1_(*k*), the asymptotic value on recovery by Φ_2_(*t*,*k*), and the rate of recovery modeled by Φ_3._

### Primary pre-specified analyses

#### Recovery of responsiveness

Given that the induction of generalized seizures by electrical stimulation may compound the recovery from general anesthesia, we expect that interval from loss to return of responsiveness following ketamine + ECT will be longer compared to the period for ketamine + sham ECT. We will determine the median and 95% confidence intervals for this measure in relation to the treatment intervention.

#### Recovery of cognition

Given that postictal suppression may be prolonged with ketamine than with etomidate (78), we hypothesize that the time needed for the return of cognition to baseline on individual sessions will be greatest for ketamine + ECT, followed by etomidate + ECT, and ketamine + sham ECT. Separate mixed-effects models will be generated based on reaction time and accuracy. We will determine the time of convergence for 95% confidence intervals for the marginal responses related to the three treatment groups. To determine the timing for the recovery to baseline, we will determine the time when the same 95% confidence intervals include 0.

#### Postictal delirium and cognitive recovery

We expect the incidence of delirium (3D-CAM) to be associated with delayed restoration of baseline function in all cognitive domains. The magnitude and significance of this relationship will be determined from the mixed-effects models for each cognitive test.

#### Comparison of cognitive recovery after ECT and isoflurane general anesthesia

We hypothesize that the time for recovery to baseline will be quicker for treatments involving ECT compared to that for the recovery from isoflurane general anesthesia. Convergence for 95% confidence intervals will be compared between treatment groups.

### Secondary pre-specified analyses

#### Recovery of cognition

Principal measures of performance are based on preliminary data (60) of the Cognition test battery. Additional measures include: PVT response speed, DSST throughput, PVT lapses, VOLT duration and accuracy, AM duration and accuracy, and DSST errors. Cognition performance measures associated with lower effect sizes will also be assessed. These include AM accuracy, VOLT accuracy, F2B reaction time, MPT accuracy, MPT duration, and F2B accuracy. Overall, we expect F2B, VOLT, and AMT to recover the slowest due to taxing of short-term memory after ketamine + ECT compared to ketamine + sham ECT.

#### Recovery in the spontaneous EEG

We will evaluate mixed-effects models to test the hypotheses that the predominance of frontal delta or theta power during passive eyes opening or occipital alpha power during eyes closure predict cognitive performance (Cognition scores) or delirium (3D-CAM scores). We will also compare these EEG spectral measures across treatment sessions to evaluate the impact of anesthetic and ECT.

#### Relationship of ECT seizure duration to return of responsiveness to verbal command

We expect that the length of ECT-induced seizures will correlate with the intervals from the loss of responsiveness to the return of responsiveness.

#### Mood and depression severity

We expect improvement in these clinical outcomes to be greatest with ketamine + ECT, followed by etomidate + ECT, and then ketamine + sham ECT.

#### Treatment satisfaction

We expect satisfaction to be greatest for ketamine + ECT, followed by ketamine + sham ECT, and then by etomidate + ECT.

## Risks and justification

Candidates for ECT are refractory to multiple medical modalities. These patients may benefit from a greater understanding of the impact of anesthetics and ECT on the severity of the underlying psychiatric illness and the recovery of cognitive function. Ketamine may improve the efficacy of ECT and recovery of cognitive function following ECT. Patients may benefit from the additional EEG monitoring in the postictal period during which non-convulsive status epilepticus is rarely manifested. Risks from exposure to ketamine include self-limited tachycardia, hypertension, hallucinations, agitation, and delusions.

## Research conduct

Treatment sessions will be conducted at Washington University School of Medicine under the general supervision of a board-certified anesthesiologist who is familiar with post-anesthetic and post-intervention care. Patients will be monitored during emergence and recovery from anesthesia by the anesthetic and nursing staff in the ECT suite as per standard care. Additionally, research personnel trained in good clinical practices will be present during the acquisition of data. Monitoring and safety will be according to the current clinical standard. Patient confidentiality will be maintained through de-identification of personal health information. Identity and linking information will be stored in a locked cabinet within the principal investigator's office, which is locked outside of business hours. Electronic data will be password-encrypted on secure servers.

Patient satisfaction will be monitored throughout the study (Supplementary Material 2). Discontinuation of study procedures will be proposed if withdrawal is in the best interest of the patient or if removal of treatment blinding is requested. In the event that different interventions are needed from those allocated, the patient will be withdrawn from the study. Participants will be given a satisfaction survey to be returned by mail following the last study session (79).

## Adverse events and premature discontinuation

The Washington University in St. Louis Human Research Protection Office (HRPO) and the Data and Safety Monitoring Committee (DSMC) will oversee the study's progression and adherence to protocol. The study was approved by HRPO on March 24, 2016. Following each intervention, the principal investigators will affirm continued involvement or withdrawal based on patient tolerance and data quality. All adverse events will be reviewed by the DSMC and reported to HRPO, following the reporting policies and procedures, and followed until satisfactory resolution. The description of the adverse experience will include the time of onset, duration, intensity, known etiology, relationship to the study, and any treatment required. The trial steering committee will be responsible for all major decisions regarding changes to the protocol. The committee will communicate these changes to HRPO and appropriate parties.

## Dissemination

The final trial dataset will be the property of the investigative team and shall not be shared without permission from the principal investigators. Dissemination plans include presentations at local, national and international scientific conferences. Every effort will be made to publish results of this trial in peer-reviewed journals. Dissemination of results to study participants and their family members will be available upon request. Updates and results of the study will be available to the public at www.clinicaltrials.gov. The trial was first registered on April 30, 2016 as NCT02761330. The first participant was enrolled on May 3, 2016.

## Discussion

This study will be the first to elaborate the time course and sequence whereby consciousness, cognition, and EEG activity recover following seizures induced for ECT. The randomized, repeated methods design of the study is powerful, as it will allow several within-patient comparisons. Specifically, the design will allow us to distinguish between the effect of ketamine anesthesia alone versus the combined effect of ketamine and ECT. Recovery of consciousness after etomidate is expected to be rapid, on the order of minutes. Therefore, the etomidate + ECT arm should provide an additional comparator condition to the ketamine + ECT arm. Intra-patient repetition of each of the three exposures over 2 weeks will help to establish the reproducible effect of each exposure on the outcomes of interest.

Past EEG studies during and after ECT show interesting correlates with clinical outcomes, including degree of increased post-ECT slowing (theta and delta activity) during the course of treatment for depression (80). However, EEG acquisition techniques have varied widely in past studies, with some studies using limited EEG electrode coverage of the bifrontal regions (45) and others using more conventional standard clinical EEG montages (38). Techniques with broad head coverage and high sensor density, such as magnetoencephalography, have already shown promise in further characterizing post-ECT changes such as increases in slow delta and theta rhythms, and decreases in faster alpha and beta rhythms (81). The use of high-density EEG in the current study offers an extension of current clinical monitoring with extensive electrode coverage over the scalp. This approach may expand the possibility of detecting meaningful EEG changes that correlate with post-ECT changes in consciousness and treatment effects.

Limitations of the study include an inability of our sample size to account for variability across medical treatment regimens. Despite our efforts to minimize subjective bias across treatment arms, patient unblinding is possible due to psychoactive effects of ketamine that are not associated with etomidate. Furthermore, the lack of pharmacologic muscle paralysis during sham ECT treatments may also contribute if muscle aches are encountered during the study. Without drug levels of ketamine, we will be unable to rule out the possibility that any prolongation in depressed cognition or consciousness is related to changes in hepatic blood flow or function during ECT compared to sham treatments. Finally, the effects of timing between treatments and of successive treatments over time may not be fully addressed by our study design.

The cognitive domains assessed through the study test battery are components of complex cognoaffective processes that may be more closely linked to activities of daily living. Further investigation will be able to address measures that integrate cognitive domains and emotional valence or account for cognitive distortions (82). While potentially more difficult to assess, these meaningful outcomes will yield a greater understanding of how patients perceive and interact with their environment.

An underlying motivation for this work is that cognitive impairments incurred over the ECT index course limit patient functionality and adherence to this important treatment modality. Comparisons of task performance before and after an index course have identified deficits in orientation (83, 84) and different forms of memory (47, 85–90). Cumulative treatments of ECT are associated with anterograde (91) and retrograde amnesia (92), primarily following the entire course regimen (40). Cognitive deficits resolve within days (40) to weeks after the index course of therapy (87, 88, 90, 93). The links between these longer-term deficits and the perturbations incurred over the shorter time scales of the proposed investigation constitute future avenues of inquiry.

We anticipate our findings to lay the groundwork for larger mechanistic studies for generating markers that reflect the efficacy and side effects of ECT across an array of psychiatric illnesses. These insights could impact our understanding of other neuromodulatory therapies. Overall, ECT remains an established and effective modality for treating refractory mental illness. Future neural markers for aiding decision-making of patients and clinicians could demystify ECT, thereby improving access, adoption, and adherence.

## Author contributions

BP, HM, AM, NF, ET, EL, MB, and MA contributed to study design; BP, HM, AM, NF, RH, ET, JS, TW, SC, EL, MB, MK, and MA contributed to the writing of the manuscript.

### Conflict of interest statement

The authors declare that the research was conducted in the absence of any commercial or financial relationships that could be construed as a potential conflict of interest.
