# Supplementary material for: Cognitive and Neurophysiological Recovery Following Electroconvulsive Therapy: A Study Protocol
Source: Front Psychiatry. 2018 May 14;9:171. doi: 10.3389/fpsyt.2018.00171 (PMC5960711; doi:10.3389/fpsyt.2018.00171)
Supplement: Supplementary file 1 [file Data_Sheet_1.PDF]

Supplementary Material 1: Self-Assessment Manikin (SAM)

How do you feel right now?

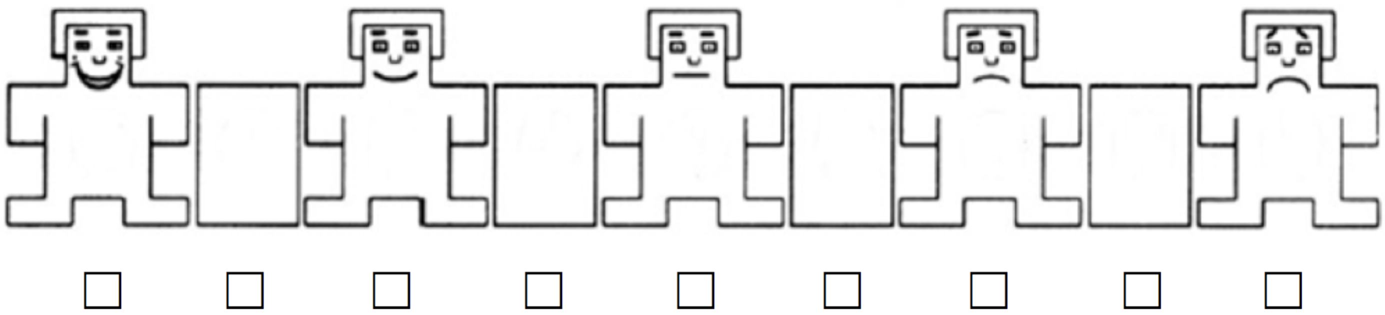

We will show the scale to the patient and read the following script:

"This scale asks how you are feeling right now. It ranges from a smile to a frown. This end of the scale (point) **means you feel completely happy, pleased, satisfied, contented, hopeful. The other end of the scale** (point) **means you feel completely unhappy, annoyed, unsatisfied, melancholic, despaired, bored. You can choose any of these points to describe how you feel right now. "**

The patient will then place a check mark in the box below the manikin row that most accurately depicts his/her mood.

Supplementary Material 2: Assessment of Patient Satisfaction

1. Did you have any side effects or problems after receiving treatments? Please indicate after which treatment these side effects occurred (check all that apply).

	1st	2nd	3rd	4th	5th	6th	Yes, but not sure when
Nausea							
Vomiting							
Confusion							
Bruising or tenderness at IV site							
Fever							
Headache							
Weakness							
Memory loss							
Agitation							
Hallucinations							
Jaw pain							
Nightmares							
Other:___________________________							

The following items refer to your experience throughout the entire study. Please rate how well you agree with each statement.

	Strongly agree	Agree	Neutral	Disagree	Strongly disagree
2. I was satisfied with my care.					
3. Overall, I felt comfortable throughout the study.					
4. I learned about consciousness and cognition by participating.					
5. I was well compensated for this study.					
6. I am satisfied with my interactions with the study team.					
7. I would participate in this study again.					
8. My condition improved after completing the treatments.					

9. What was the most physically uncomfortable or distressing part of the study?
    - Consent
    - Screening questionnaires (performed in the days or weeks before treatment)

EEG cap
Morning before treatment
Recovery from treatment
If yes, please indicate which (check all that apply):
  1st
  2nd
  3rd
  4th
  5th
  6th
No period was particular uncomfortable or distressing
Other: ____

10. How physically uncomfortable or distressing was the experience selected in the previous question?
    Extremely
    A lot
    A little
    Not very
    N/A

11. What did the study team do well?
    ____
    ____

12. Which aspects could the study team improve?
    ____
    ____

13. Additional comments:
    ____
    ____
    ____
